# Oxytocin and cardioprotection in diabetes and obesity

**DOI:** 10.1186/s12902-016-0110-1

**Published:** 2016-06-07

**Authors:** Marek Jankowski, Tom L. Broderick, Jolanta Gutkowska

**Affiliations:** Cardiovascular Biochemistry Laboratory, CRCHUM (7-134), Tour Viger, 900 St-Denis St., Montreal, Quebec H2X 0A9 Canada; Department of Medicine, Faculty of Medicine, University of Montreal, Montreal, Canada; Department of Physiology, Laboratory of Diabetes and Exercise Metabolism, Midwestern University, Agave Hall, office 217-B, 19555 North 59th Avenue, Glendale, AZ 85308 USA

**Keywords:** Oxytocin, Heart, Cardiomyocyte, Differentiation, Stem cells, Cardioprotection

## Abstract

Oxytocin (OT) emerges as a drug for the treatment of diabetes and obesity. The entire OT system is synthesized in the rat and human heart. The direct myocardial infusion with OT into an ischemic or failing heart has the potential to elicit a variety of cardioprotective effects. OT treatment attenuates cardiomyocyte (CMs) death induced by ischemia-reperfusion by activating pro-survival pathways within injured CMs in vivo and in isolated cells. OT treatment reduces cardiac apoptosis, fibrosis, and hypertrophy. The OT/OT receptor (OTR) system is downregulated in the *db/db* mouse model of type 2 diabetes which develops genetic diabetic cardiomyopathy (DC) similar to human disease. We have shown that chronic OT treatment prevents the development of DC in the *db/db* mouse. In addition, OT stimulates glucose uptake in both cardiac stem cells and CMs, and increases cell resistance to diabetic conditions. OT may help replace lost CMs by stimulating the in situ differentiation of cardiac stem cells into functional mature CMs. Lastly, adult stem cells amenable for transplantation such as MSCs could be preconditioned with OT ex vivo and implanted into the injured heart to aid in tissue regeneration through direct differentiation, secretion of protective and cardiomyogenic factors and/or their fusion with injured CMs.

## Background

OT was the first peptide hormone to have its structure determined and the first to be chemically synthesized in a biologically active form [1]. OT acts as a brain neuromodulator and central nervous system (CNS) regulator functionally different than the second neurophyseal hormone, arginine-vasopressin (AVP). Both hormones are mainly produced in magnocellular cells of hypothalamic parvocellular paraventricular nucleus and supraoptic nucleus neurons [2]. OT is released locally in the brain and systemically into the circulation. OT and AVP play an important role in many physiological functions through GPCR (G-protein-coupled-receptor) signal transduction. Like AVP, OT is a disulfide-bridge cyclic nonapeptide but contains neutral amino-acids at position 3 and 8 (isoleucine and leucine, respectively). The difference in polarity of these residues, compared to AVP, is believed to enable OT to interact predominantly with its specific receptor subtype [3]. In humans and other mammalian species, OT and AVP target the OTR (OT receptor) and the three vasopressin receptors V1aR, V1bR and V2R. Whereas AVP binds to the OTR and AVP receptors with almost identical affinity, OT has a highest affinity for OTR and lower for AVP receptors. Study on transfected cell lines [4] revealed that OT had a high affinity for the OTR (K(i) expressed as mean = 6.8 nmol/L) and bound, to some extent, to the AVP V1aR (K(i) = 34.9 nmol/L). AVP displayed higher affinities for AVP V1a, V1b and V2 receptors (K(i) = 1.4, 0.8 and 4.2 nmol/L, respectively) than for the OTR (K(i) = 48 nmol/L).

A single OTR can activate multiple second-messenger pathways and the OTR is found in many tissues, including the kidney, ovary, testis, pituitary, heart, vascular endothelium, adipocytes, myoblasts pancreatic islets and regulatory T cells [3]. The heart, especially the right atrium is also one of the main sources of OT [5].

Gene deletion showed that OT is essential for milk secretion and regulates maternal behavior, social recognition, sexuality, memory, pair bond formation [6], and obesity [7–14]. OTR ligands with high specificity over the related AVP receptors are currently available and several patents propose their therapeutic utility in numerous applications, e.g. increasing lactation, acting against preterm labor, breast cancer, modulating immune system, osteoporosis, and autism-related disorders (reviewed in [15]). Potential novel application of OT include treatment of autism, schizophrenia, depression, social anxiety, and Prader-Willi syndrome [16]. Because OT reduces glycemia [13], the potential indications for clinical use include diabetes (patents: US20140066373, D. Cai; WO 2011/14505, F. Rohner-Jeanrenaud and N. Deblon), prediabetes, and insulin resistance. In animal studies, OT has been shown to reduce food intake and produce fat weight loss and OT antagonist reverses this effect as recently reviewed [12, 17, 18].

## Oxytocin signaling in the heart

**Fig. 1:**
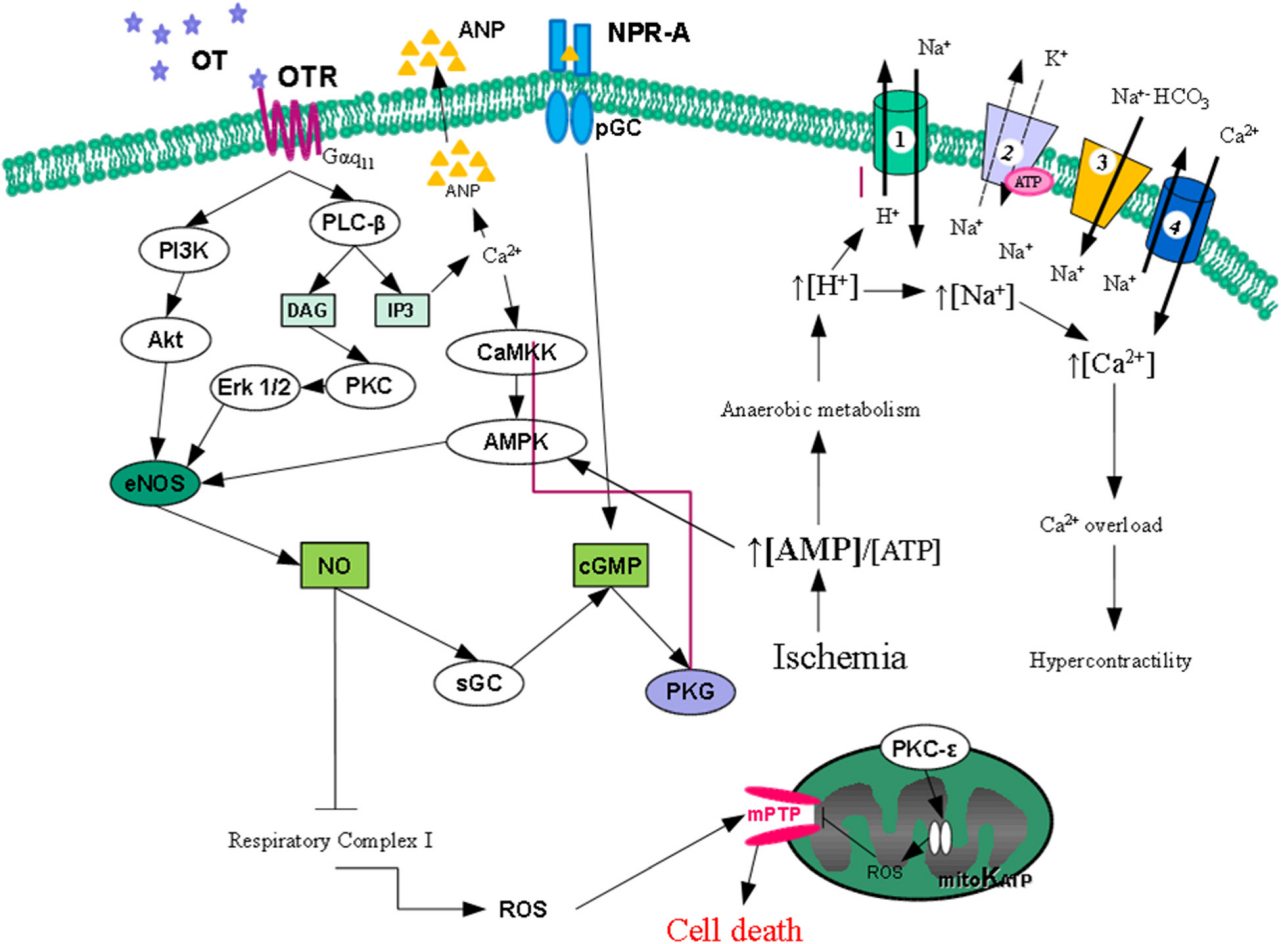
Proposed cardioprotective signaling of OT during ischemia reperfusion (modified from reference [19]). OT acts via its G protein coupled receptors (GPCR). OT triggers the PLC-β and PI3K pathways to stimulate NO production, the regulation of ionic pumps and subsequent inhibition of the mitochondrial permeability transition pore. OT also stimulates ANP release, which in turn binds to NP receptor A to also inhibit mPTP opening. OT: oxytocin; OTR: OT receptor; PLC-β: phospholipase C type β; DAG: diacylglycerol; IP3: inositol-3-phosphate; PKC: protein kinase C; Erk1/2 : Extracellular regulated kinase 1 and 2; PI3K phosphatidylinositol-3-kinase; eNOS: endothelial nitric oxide synthase; NO: Nitric oxide; sGC: soluble guanylate cyclase; pGC: particulate guanylate cyclase; cGMP: cyclic guanosine monophosphate; PKG: protein kinase G; ROS: reactive oxygen species; mPTP: mitochondrial permeability transition pore; mitoKATP: mitochondrial ATP-dependent K^+^ channels; CaM: Ca^2+^- Calmodulin; CaMKK: CaM kinase kinase; AMPK: cAMP-activated protein kinase; NPR-A: Natriuretic peptide receptor type A. 1. NHE: exchanger Na^+^/H^+^ present on cell membrane; 2. Na^+^/K^+^ ATPase pomp; 3. Co-transporter Na^+^/bicarbonate; 4. NCE: exchanger Na^+^/Ca^2+^

OT is recognized as a cardiovascular hormone with cardio-protective effects [19]. OT and OTR are synthesized in the human and rat heart [20, 21] and OT exerts cardio-protection either directly or via stimulation of mediators such as the natriuretic peptides (NPs) [20, 22] and nitric oxide (NO) [23]. Both these mediators activate the cyclic guanosine 3′,5′-monophosphate/cGMP-dependent protein kinase (cGMP/PKG), following activation of soluble or particulate guanylate cyclases (sGC, pGS), respectively. The cardiovascular effects of OT include natriuresis and lowering of blood pressure, negative cardiac inotropy and chronotropy, NO-induced vasodilatation and endothelial cell growth. The cardiac OT system has been shown to regulate both cardiac cell survival pathways and provide protection against ischemic heart injury [24, 25]. It has been recently suggested that therapies enhancing the cardiac OT/OTR system prevent CMs apoptosis following an ischemia-reperfusion (IR) insult [26]. CM death can be prevented or attenuated by conditioning of the heart, with OT treatment initiated either before or after an ischemic insult [27]. Apart from PKG activation, NO has also been proposed as a cytoprotective factor due to the attenuation of reactive oxygen species (ROS) production during reperfusion caused by the reversible inhibition of mitochondrial respiratory complex I by S-nitrosation [28]. Proposed OT signaling in CM is illustrated on Fig. 1. ANP is an established cardioprotective peptide because of its ability to inhibit the release of renin and sympathetic nerve activity, and the synthesis of aldosterone. ANP also ameliorates endothelial function and decreases fibrosis, inflammation and apoptosis in myocytes which are associated with LV remodeling [29]. Furthermore, ANP activates the reperfusion injury salvage kinase (RISK), which has been shown to mediate ischemic preconditioning and postconditioning. OT in physiological concentrations (~10 nM) prevented the development of newborn and adult rat CMs hypertrophy exerted by ET-1 and Ang-II by several mediators such as PI3K/ERK1/2/ANP-cGMP/NFAT signalling [30].

## OT and cardioprotection

The role of OT in cardioprotection was recognized more than 50 years ago by Melville and Varma [31] in experiments executed on rabbits. They observed that the marked ST-T depression on an electrocardiogram, resulting from hypoxia or ergometrine administration, was reduced after intravenous OT injection (1 unit/kg body weight). Interestingly, they also observed that experimental ST-T changes induced by AVP were abolished by OT co-treatment. Although the authors postulated a metabolic action of OT enhancing oxygen consumption in cardiac muscle, the presence of OTR in the heart was discovered 35 years later [20]. We have recently demonstrated that OT treatment reduces lethal reperfusion injury of H9c2 cardiomyoblasts and inhibits ROS production in cells exposed to hypoxia [25]. This protection of cell viability was evoked through the intact OTR because in cells with reduced OTR levels due to the siRNA-mediated knockdown, treatment with OT in I-R caused an elevated cell death compared to untreated control cells [25]. It is possible that in the absence of OTR, OT triggers deleterious signaling via the AVP receptor. We would thus expect that AVP signaling is detrimental in this context, and OT is favorable. However this statement may not be accurate in view of the recent study by Phie et al. [32]. They demonstrated that in the rat model of Ang II-induced hypertension, chronic co-treatment with OT failed to prevent the increase of blood pressure and LV hypertrophy. The authors observed enhanced end-organ renal injury in rats receiving a combination of OT and Ang II. Indeed, interaction of Ang II and AVP/OT in the kidney can influence cardiovascular homeostasis by several mechanisms involving V2R signaling [33].

Several studies have presented the possibility that OT can serve as a drug for the treatment of ischemic cardiac disease [19, 34]. OT was also shown to have transient negative inotropic and chronotropic effects on perfused isolated dog right atria mediated by NO production and acetylcholine release at cardiac parasympathetic postganglionic neurons [35]. Ondrejcakova et al. [36] study on isolated Langendorff-perfused rat hearts showed that the elimination through electrical stimulation of the negative chronotropic effect of OT prevented its cardioprotective action. Furthermore, the authors revealed that perfusion of the hearts with OT before ischemia resulted in significant reduction of the infarct size [36]. The impaired cardiac work and down-regulation of the OT/OTR system following MI could be reversed by administration of OT given before the onset of ischemia and during one week of reperfusion in a rat model of MI [24]. In the same study, we have shown that treatment with OT reduced the expression of pro-inflammatory cytokines (TNFα, IL-1β and IL-6) and reduced immune cell infiltration (especially of neutrophils) [24].

Work by Alizadeh et al. [34] showed that the reduction in infarct size in the anesthetized rat heart induced by a pre-treatment with OT (0.03 μg/kg i.p.) 25 min prior to ischemia was eliminated by the co-treatment with an inhibitor of the opening of mitoKATP channels, which have been proposed as the end-effectors of cardioprotective ischemic pre- and postconditioning. In the study of Ondrajeckova et al. [37] on perfused hearts isolated from male Wistar rats treated chronically with OT, the cardioprotection was linked with specific phosphorylation (activation) of p38-MAPK and Akt kinase, an increase in phosphorylated Hsp27 and an elevation in atrial natriuretic peptide (ANP) levels in left ventricular heart tissue. Correspondingly, in H9c2 cells, the increased viability achieved by OT treatment was decreased in the presence of Wortmannin (the Pi3K-Akt pathway inhibitor), the cGMP-PKG inhibitor KT-5823, the sGC inhibitor ODQ and the pGC antagonist A71915 [25]. Confocal microscopy demonstrated that following OT treatment p-Akt appeared to accumulate around the cell nuclei and co-localized with the mitochondrial marker Cox IV. It is thus possible that OT stimulates protection against ROS insult by the formation of signalosomes. This hypothesis is derived from our experiments showing that OT stimulation causes intracellular signaling involving Pi3K, Akt and eNOS [38], known as the canonical factors of signalosomes derived from GPCR [39].

We have also observed the paradox that OT treatment stimulates the production of moderate levels of ROS whereas OT inhibits excess ROS produced as a consequence of ischemia [25]. The high levels of ROS are detrimental for CMs but moderate levels of ROS function as signaling molecules for cardioprotection by activating protein kinases such as Pi3K/Akt within and outside the mitochondria [40], as well as the ERK1/2, MAPK p38 and/or JAK/STAT prosurvival signalling cascade [28, 41].

## Obesity and OT treatment

Several key metabolic effects of OT have been reported such as anti-inflammatory activity, wound healing, antioxidant activity and increase of glucose uptake in cardiac and stem cells [19]. The adult male OTR-deficient mice [14] and both male and female OT knockout mice [42] express a mild obese phenotype. Zhang et al. [43] have shown that C57BL/6 mice fed a high fat diet (HFD) became obese and showed reduced plasma OT levels. Lowered OT levels in the circulation are also reported in human diabetes type 1 and 2 [44, 45]. Chronic OT administration protected against HFD-induced obesity in rodent models [7, 8, 46]. A recent study of Blevins et al. [47] indicates that chronic that a chronic increase (≈21–26 days) of CNS oxytocin signalling not only prevented weight gain induced by HFD but also effectively reduced already established diet-induced obesity. OT also decreased the genetic-induced obesity observed in Zucker rats [48] and *db/db* mice models [49], which bear a mutation in the leptin receptor gene.

OT has many positive metabolic effects based on remarkable changes in glucose metabolism, lipid profile, and insulin sensitivity after OT administration. OT dose-dependently reduces food intake in animal and human studies (reviewed in [17]). Importantly, OT treatment improves eating behaviors such that hedonic hyperphagia is reduced and does not interfere with normal hunger and weight regulation [17, 46, 50, 51]. The *ob/ob* mouse represents a close counterpart to the human condition of severe obesity, but unlike *db/db* mice, exhibit leptin deficiency from a mutation in the *ob*-gene [52]. Ironically, a recent study revealed that OT treatment of *ob/ob* mice worsened glycemic control, likely from an increased production of corticosterone and stimulation of hepatic gluconeogenesis [50]. The body weight gain-reducing effect was limited to the fat mass only, with decreased lipid uptake, lipogenesis, and inflammation, combined with increased futile cycling in abdominal adipose tissue. Correspondingly, several clinical trials (UKPDS33, ACCORD, ADVANCE, and VADT) demonstrated that intensive glycemic control fails to prevent cardiac complications in diabetics or have even increased cardiovascular mortality [53]. This calls for the development of new strategies capable of preserving heart function in diabetes.

OT intranasal delivery, secure for the treatment in humans, appears to effectively enable OT to enter the CNS in mice, rats, nonhuman primates and humans within 30–35 min post-treatment [54–56]. Intranasal OT administration increases OT levels also by stimulation of endogenous synthesis [54, 57], resulting in the prolongation of the OT half-life and efficacy [58]. This mode of delivery may also increase OT levels in the circulation if the intranasal delivery device did not sufficiently target the cribiform plate [59]. In a recent study, intranasal OT administration in patients receiving 24 units 4×/day (1 unit ~ 1.7 μg; 1.77 μg/kg body weight/day) over a period of eight-weeks reduced obesity and reversed prediabetic changes [13]. We found that increases in both energy expenditure and brown adipose tissue volume contribute to the decrease of body mass of OT-treated *db/db* mice [49].

## The role of exercise in obesity and diabetes

Weight loss improves cardiac function in obesity [60] and especially exercise training reduces events of HF [61]. There is substantial evidence to support the value of regular exercise training in patients with obesity and diabetes [62]. Exercise improves overall cardiorespiratory fitness and decreases the risk of developing cardiac and vascular injury [63]. Exercise training is also associated with an increase in peripheral glucose utilization and reduction in white adipose tissue (WAT) content. WAT is source of leptin, which acts on its hypothalamic receptors to regulate energy use and induce satiety [64]. WAT mRNA expression is increased in human obesity as well as in relevant rodent models [65, 66], and chronically elevated blood levels of this peptide is known to induce leptin resistance [67], leading to a loss of appetite suppression and decreased energy production. Further, epidemiological studies indicate that plasma hyperleptinemia is an independent predictor of CV events [68]. Elevated WAT promotes the secretion of monocyte chemotactic factor-1 (MCP-1), which promotes infiltration of macrophages into WAT [69], increased expression of TNF-α and IL-6, and decreased expression of adiponectin. This abnormal cytokine pattern is known to attenuate insulin signaling responses in tissues, leading to insulin resistance [70]. In diet-induced obesity, running activity decreases the expression of mRNA for leptin in both visceral adipose tissue and WAT [71, 72]. However, the effects of regular exercise on WAT and leptin content in plasma of humans are inconsistent, largely due to differences in the exercise program, duration of intervention, pre-exercise body and fat mass index, and whether exercise is combined with diet restriction [73].

## The cardiac OT/OTR system and exercise

The cardiac OT system is downregulated in DC [74], MI [24], and hypertension [75] suggesting that OTR deficiency magnifies these pathologies. Lowered OT plasma levels are also linked to increased sensitivity of cardiovascular system to stress [76]. Interestingly, down-regulation of the OT/OTR system (which includes natriuretic peptides (NPs) ANP, BNP) in the LV of ovariectomized rats can be effectively prevented by regular exercise training [19]. Indeed, hypothalamic and LV mRNA levels of OT, OTR, and NPs were restored back to normal levels after 8-weeks of moderate intensity exercise training on a treadmill. We have investigated the effects of exercise training in the *db/db* mice, a mouse model of diabetes and obesity used to study DC. These mice display genetic mutations in leptin receptor, resulting in a diabetic profile induced by hyperphagia. We have demonstrated that exercise training failed to upregulate of the OT/OTR system in hearts of *db/db* mice. After eight weeks of moderate intensity treadmill running, mRNA expression of the OT/OTR system in *db/db* hearts remained low and unchanged from pre-training levels [74]. Expression of ANP and BNP was similarity low and even further decreased after exercise training, and training had no effect on the extent of obesity and blood glucose concentrations [72], suggesting that the hyperglycemic state may contribute to reduced expression of OT and NPs. This hypothesis was suggested earlier by Kobayashi et al. [77] indicating that acute hyperglycemic conditions and streptozotocin-induced type 1 diabetes reduced the expression of GATA4, a transcription factor associated with the synthesis of structural and cardioprotective genes, including OT/OTR and NPs [78]. Hearts from *db/db* mice exhibited low mRNA and protein levels of GATA4 [79], and exercise training was beneficial in stimulating GATA4 protein expression, albeit in the absence of the OT/OTR system. Degradation of GATA4 by hyperglycemia is induced by ROS and ubiquitination by ubiquitin-proteaosomes [80], and a benefit of exercise may be related to reduced expression of E3-ubiquitin ligase MURF1 [81]. The possibility exists that GATA4 is not required and that expression of another transcription factor, such as GATA6 [82], is involved in the synthesis of OT and NPs. The role of GATA4 and exercise training on the OT/OTR system is further complicated by our recent findings showing that expression of this transcription factor is not altered by the effects of hyperglycemia or obesity in hearts of *ob/ob* mice [83]. Eight weeks of spontaneous running in *ob/ob* mice increased cardiac mRNA expression of GATA4, but disrupted glucose and triglyceride regulation. Consistent with the gene profile observed in *db/db* mice after exercise training, expression of OTR, ANP, BNP, and C-type NP was reduced. One important aspect that arises from this *ob/ob* study is whether leptin is key to this aberrant synthesis of the OT/OTR system. In addition to its well-established role in appetite control, leptin is known for stimulating running activity in mice [84].

## Normalization of OT plasma levels and upregulation of the cardiac OT/OTR system protects the heart against DC

OT treatment in four-week old *db/db* diabetic mice for a period of 12 weeks improved body parameters, metabolic parameters (including insulin resistance) and protected against systolic and diastolic cardiac dysfunction, ROS production, apoptosis and fibrosis [49]. OT treatment prevented the reduction in cardiac *ANP* and *BNP* gene expression in *db/db* mice as well as *Gata4,* the transcription factor of genes required in the maintenance of cardiac structure and function [85]. BNP treatment in *db/db* mice also reduced CM hypertrophy and increased local OT/OTR expression [82]. OT treatment resulted in activation of Akt-1 in cardiac cells which targets an array of diverse cellular functions, including stimulate NO production, protein synthesis, energy metabolism, and cellular survival [86]. OT also increased the activity of AMP-activated protein kinase (AMPK) and induced the expression of cardioprotective genes [38]. OT treated *db/db* mice displayed decrease in expression of cardiac inflammatory markers, including NF-kB, the IL-1β and IL-6. The marked improvement of the cardiac work and structure, measured by echocardiography, was achieved by OT treatment in spite of a modest reduction of glycemia [49]. We found that increases in both energy expenditure and brown adipose tissue volume contribute to the decrease of body mass of OT-treated *db/db* mice [49]. A recent study reports the contribution of the OT system in the alleviation of obesity and reduction of glycemia by retinoic acid treatment in leptin-deficient, *ob/ob* obese diabetic mice [87]. These results suggest the usefulness of OT as a therapeutic alternative because it bypasses the defective insulin-signaling pathway(s) in subjects with type 2 diabetes with obesity.

The mechanisms responsible for cardiac protection by OT against DC are not yet understood. It is unclear whether OT acts via a number of organs and tissues, or specifically enhances OT/OTR signaling in cardiac cells.

## Oxytocin treatment provides a method of regenerating cardiac tissue

Some evidence indicates that the heart, long considered to be a terminally differentiated organ, contains CM-like cells which undergo mitosis, as reported in patients with acute myocardial injury [88]. These cells are defined as cardiac progenitor cells (CPCs) and may contribute in cardiac reparative processes [89]. Diabetes exerts a detrimental effect on the proliferation and survival of CPCs in both humans and animal models of diabetes [90]. Similarly, aging and diabetes mellitus also have been shown to correlate negatively with other stem cell types such as MSCs [91] and endothelial progenitor cells (EPC) [92].

Oxidative stress induces senescence of CPCs in diabetic cardiomyopathy [93]. It is suggested that the premature aging of cardiac stem leads to the development of HF [94]. The loss of cardiac cells is accompanied by a decrease in cardiac muscle mass, chamber dilation, and impaired ventricular function [95]. Recent reports suggest that senescent CPCs from a diseased heart can be activated and reverted to the “young” phenotype by treatment with factors that change cellular signaling [96]. OT stimulates the proliferation of cardiac stem cells [97, 98], and also their differentiation to CMs and ECs [19]. The expression of the OT/OTR system correlates with youthful cardiac phenotypic characteristics [21], and it is possible that diabetes-related changes in viability, morphology and proliferation can be reduced by OT treatment.

MSCs are able to hone in on the injured heart, to engraft into damaged blood vessels, to differentiate into cardiac cells, and to exert a paracrine effect by the local release of vascular growth factors and cytokines [99]. Recent studies have shown that direct injection with OT-treated MSCs into the rat heart after ischemia-reperfusion injury improves their engraftment rate and results in an enhanced cardioprotective effect via increased transmigration activity to the injured zone, the upregulation of matrix metalloproteinase-2 mRNA, the integration of MSCs into the myocardium [100]. OT stimulates in these cells the production of paracrine anti-fibrotic and anti-inflammatory factors [98]. For improving survival, proliferation, and differentiation MSC in diabetic conditions either in vitro or after transplantation in pre-clinical models, Kim et al. proposed OT as a priming reagent restoring the angiogenesis activity of MSC [101].

## Conclusions

**Fig. 2:**
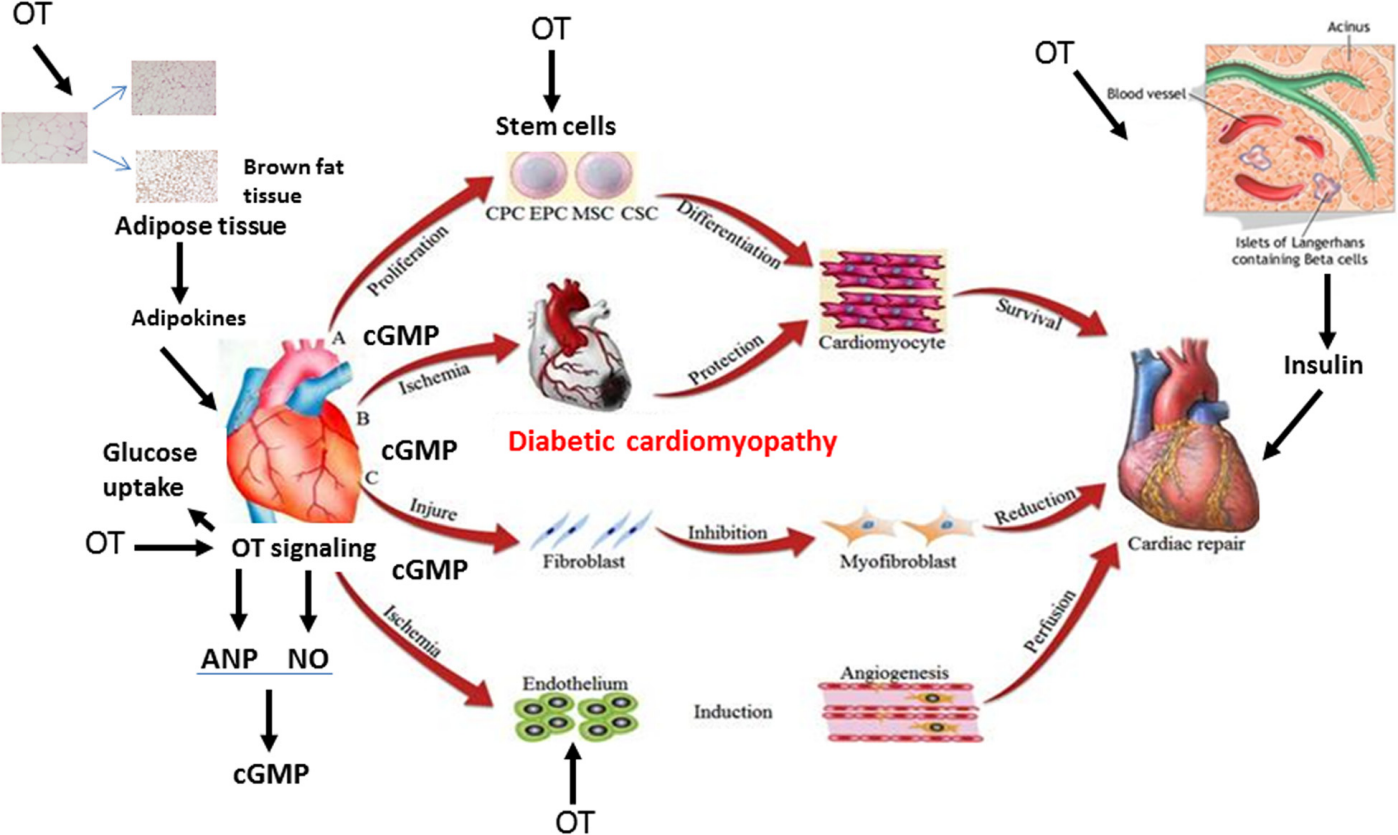
Oxytocin (OT) treatment in diabetes protects against diabetic cardiomyopathy The mechanism involve direct OT effect on the heart and release of atrial natriuretic peptide (ANP) and nitric oxide (NO) as well as their second messenger cyclic guanylate cyclase (cGMP). OT reduces fat deposits through decrease of adipocytes size and brown fat production. Adipocytes release adipokines to the circulation which are beneficial for the heart. In pancreas OT stimulates insulin release

Several investigations have clearly established the role of OT in lowering of body weight by mechanisms involving increased energy expenditure, reduced adiposity and food intake. In models of diabetes and obesity as well as in human diabetes, OT deficiency has been reported. Improvement in body weight and composition can be obtained by central, peripheral and intranasal OT administration. In addition, an OT effect as a prosocial hormone may provide additional benefit in the treatment of complex diseases such as diabetes and metabolic syndrome. Preclinical studies on animal models have demonstrated that OT limits myocardial ischemia and reperfusion injury, independent from its glucose-lowering effect. The OT-mediated cardioprotection include activation of the NPs and NO both increasing formation of cGMP in the heart, activation of AMPK and by inhibition of excess of ROS produced as a consequence of ischemia. The potential OT benefit in diabetic cardiomyopathy is illustrated on Fig. 2. Exercise training increases the OT/OTR system in the ovariectomized-rat, but the role of exercise on this system in obesity and diabetes remain poorly understood. The major clinical consequence of diastolic dysfunction is exertional dyspnea, which impedes the capacity of diabetic individuals to perform exercise, an important aspect of diabetes management, particularly in the obesity [102]. Considering the efficacy of intranasal OT delivery in stimulating the synthesis of central and peripheral OT, and in reducing obesity and hedonic eating habits, investigation into the role of combined intranasal OT treatment and exercise training are warranted. Consequently, treatment with OT might potentially improve cardiovascular outcome in patients at risk for heart failure especially in association with obesity and diabetes.
